# Adrenalin and Cocain for Local Anesthesia

**Published:** 1904-04-15

**Authors:** Fritz Hartwig

**Affiliations:** Vienna


### ADRENALIN AND COCAIN FOR LOCAL ANESTHESIA.

BY DR. FRITZ HARTWIG, DENTIST, VIENNA.

Extract from "Wiener Zahnaerztliche Monatsschrift," June, 1903.

Up to the present time cocain is still the most important agent employed for local anesthesia, as may be gathered from the literature relating to the subject. All attempts to replace it by less toxic substances have failed, because no agent has shown the deep anesthetic quality of Cocain. Even tropacocain, much recommended in difficult cases, does not give a sufficiently deep anesthesia. For a number of years many investigators have tried in various ways to improve the methods of local anesthesia and render them harmless. In order to understand the lines on which these investigations should be carried out, it is necessary to ascertain on what the depth of anesthesia depends.

(1) On the kind of agent employed: Cocain produces a deeper anesthesia than eucaine; eucain is preferable to tropacocain, etc.

(2) On the pressure with which the solution employed is sent into the tissues; the greater the pressure with which the solutions are injected, the greater their anesthetizing value.

(3) On the concentration of the solution employed.

(4) On the distribution of the solution in the tissues, and also on the anesthetic agent coming into contact with as many sensitive nerve-endings as possible.

(5) On the total amount of blood in and the vitality of the tissues.

The danger of intoxication increases:

(1) With the specific toxicity of the agents employed.

(2) With the concentration of the solution employed.

(3) With the capacity for and speed of the absorption.

There is only one method of intensifying local anesthesia which has not yet been tried systematically, namely that which has reference to the quantity of blood in the tissues to be anesthetized. It was demonstrated long ago that the local action of cocain was increased by the use of Esmarch's bandage, its toxicity being diminished. But this method is not applicable to the jaws and, therefore, could not be employed in dentistry. Only recently we have been enabled to substitute for the mechanical action of Esmarch's bandage the analogous physiological action of preparations from suprarenal glands, especially adrenalin chloride, and for this reason Lermoyez has called it "the Alkaloid of Esmarch's bandage."

Adrenalin is the isolated active principle of the suprerenal gland. The preparation most widely known and used in commerce is adrenalin chloride, manufactured by Parke, Davis & Co. It is a yellowish white, crystalline substance, stable, soluble in cold water, more so in hot water, readily soluble in acids and alkalies. As it is difficult to prepare a good, durable and clear solution extemporaneously, the above firm put a solution of it on the market in the following form:

| | |
|---|---|
| Adrenalin Chloride | 0.1 |
| Natrii chlorat | 0.7 |
| Chloretone | 0.5 |
| Distilled water | 100.0 |

This solution (when kept from light and air) retains its properties for four or five months, owing to the chloretone. Adrenalin possesses a marvelous power of contracting the involuntary muscular fibers and all organs consisting of them, especially arteries and capillaries; it also increases the action of the heart by directly influencing its nerve supply. Solution adrenalin chloride 1:10,000 may be diluted to 110,000 and even then it promptly produces the above mentioned physiological reaction on the involuntary muscular fibers and consequently also a rapid anemia of normal and diseased mucous membranes and the more deeply situated tissues. The dilution must be made with distilled water, or, better, with normal salt solution. It should be kept in a dark bottle. Exposed to daylight and mixed with tap water the diluted solution, after a short time, assumes a reddish color and soon becomes inefficacious. Even in dark bottles this weak solution assumes after some time a reddish color and does not possess as much power as a fresh solution. But when the preparation is exposed to the air it becomes dark brown, forming a deposit, and is inactive. The aqueous solution of adrenalin chloride is not decomposed by boiling and can thus be easily and surely sterilized (it is destroyed only at a temperature of 230 degrees Farenheit).

I first prepared solutions of adrenalin chloride in distilled water—one milligram in the required quantity of liquid necessary for injection into the gums, *i. e.*, 1 to $1\frac{1}{2}$ syringefuls (a syringe containing four gm.), and I added to these solutions cocain in various quantities, in order to make up a mixture containing sufficient cocain for complete anesthesia. In this way I verified the fact already known, that the additions of adrenalin to cocain greatly increases the anesthetic property of the latter, and that weak solutions act better than concentrated ones minus the adrenalin.

Therefore it was possible in a great many instances to use even as little as two percent. cocain solutions and to obtain almost the same effect as with two percent cocain solutions without adrenalin. But I ascertained that very weak solutions of cocain do not give a sufficiently deep anesthesia in difficult cases, and at the conclusion of my experiments I found it most practicable to use a five tenth percent solution ordinarily, and for grave cases, especially in acute periostitis, a one to one and five tenth percent cocain solution with addition of one milligram adrenalin.

The formulas for these two principal solutions are:

For a one-half percent solution of cocain use:

| | |
|---|---|
| Solution adrenalin chloride (1:1000) | 10.0 |
| Cocain | 0.5 |
| Distilled water | 100.0 |

For a one percent solution use:

| | |
|---|---|
| Solution adrenalin chloride (1:1000) | 10. |
| Cocain | 1. |
| Distilled water | 10. |

For a one and one-half percent solution use one and one-half grains of cocain in the above formula.

I always prepare these solutions as required, the powdered cocain being divided into appropriate doses beforehand. By means of the syringe I measure the exact amount of the adrenalin solution necessary and also the quantity of liquid for dissolving the cocain (distilled water or normal salt solution). The quantity of these solutions employed for one extraction is four to eight c.c. In the five-tenth percent cocain-adrenalin solution there is two-hundreds to four-hundreds of cocain chloride, in the one percent solution four-hundreds to six-hundreds. In spite of large doses of cocain I have never experienced any toxic effects from its employment. This may be explained in two ways.

(1) Because of the strong dilution of the solution, and by the addition of adrenalin. The latter preparation has, as mentioned above, the power of contracting the vessels owing to its action upon the involuntary muscles, thus rendering the area of injection almost bloodless (Dr. Braun, Langenbeck's Archiv, 69). The flow of blood being lessened, it is followed by a great decrease of the functional activity of the injected tissues and, on the other hand, as a consequence of this, cocain is immediately decomposed and is thus prevented from being absorbed, as the action of adrenalin lasts for some considerable time (one to two hours). As a result the local action of cocain becomes very marked because it has been almost entirely decomposed before it has had time to be absorbed.

Having ascertained these facts, I began to employ this adrenalin-cocain mixture to produce local anesthesia when extracting teeth.

Altogether this mixture was used in about 550 cases—300 in my private practice and 250 at the general Polyclinique of Vienna.

As the ultimate success of this method depends upon the management of the above details, it is necessary for me to describe with exactness my method of subgingival injection:

The quantity of the injected solution varies from one-half to one and one-half syringefuls per tooth. The injections were made after Schleich's infiltration method by means of from six to eight punctures on both the buccal and lingual sides of the tooth.

The injection was made into as large an area as possible around the tooth, and in this way it was noticed that the greater the pressure of the injection the more perfect the anesthesia. But, even with imperfect infiltration, anesthesia was deep, and sufficient for all ordinary operative work, and experience proves that this method is preferable to ordinary cocain anesthesia.

Should any of the solution be spilt, the patient should rinse his mouth until all of the bitter taste has disappeared.

Extraction is made immediately or shortly after an injection.

The results obtained by this process were simply wonderful, the depth of local anesthesia being much greater than anything attained previously. Many patients were surprised at the extraordinary painlessness, never before met with, and very often they doubted whether the operation was over or not.

Especially marked were the results with very nervous, frightened and hysterical patients, under other circumstances the subjects of wearisome and otherwise extremely painful operations It was also very remarkable that the anesthesia penetrated deeply into the jaw-bone, thus allowing the most difficult extractions to be performed without pain, which would have been accompanied with at least considerable discomfort if another anesthetic had been employed. It very often happened that patients astonished at the absolute painlessness of the operation, wished other decayed teeth to be removed at the same sitting.

*But more remarkable were the results obtained with adrenalin cocain in acute and chronic periostitis, including all stages from coarse infiltration to alveolar abscesses.*

Altogether 150 cases of periostitis were treated with one to one and one-half percent adrenalin-cocain solutions. Extraction was carried out amost painlessly in all cases and with the deepest anesthesia; in about twenty of these cases infiltration could not be properly performed on account of loose cellular tissue, and the anesthesia was imperfect. However, very little pain was experienced. *To achieve these results an injection must be made according to the method suitable for the purpose,* which I will describe: I use a one to one and a half percent adrenalin-cocain mixture, according to the amount of pain and difficulty of the case. I introduce the syringe *invariably* on the healthy side of the jaw, where the periosteum is not diseased. The injection is made with great pressure till the tissues become white. The infiltration is continued slowly, progressing from the periphery of the diseased tooth to the affected area of the periosteum and of the jaw, whether it is inflamed or already ulcerated. This injection causes no pain contrary to the experience obtained under ordinary methods of cocain anesthesia. The excruciating agony, which often exists and is severe enough to drive the patient mad, ceases at once after an injection into the affected area. The extraction is performed three or four minutes after the injection, under the deepest anesthesia.

In no cases was pain felt afterward, or it was insignificant, if, after extraction, the alveoli were wiped out with a pledget of cotton wool soaked in carbolic acid or chloride of phenol.

There was no hemorrhage whatever in a great many instances; and in many cases it was so insignificant as to cease in thirty to sixty seconds. *Secondary hemorrhage, much feared by many authors as an after-effect of adrenalin, did not occur in any of the cases under observation. Neither*

*was there arrest in the healing processes observed.* Rarely edema or necrosis which could take place after dental operations under the following circumstances:

(1) When the buccal mucous membrane is injected.

(2) When tissues have been injured during operation.

(3) When the ingredients used for injection or treatment of the wound are of an irritating character.

The third class of cases does not occur with adrenalin-cocain solution. Neither is a completely aseptic operation in the mouth possible, nor an antiseptic state after extraction; injuries to the tissues are also unavoidable in troublesome extractions. Therefore edema does not occur if local anesthesia be employed as is the case with operations in the mouth without it.

During an operation no ill-effects were observed, particularly no intoxication from cocain, as may be the case with other methods of anesthesia.

The only disturbances noticed were a violent beating of the heart (pulse-rate sometimes up to 120) and a constricted feeling in the chest. But these symptoms only occurred in a few cases of nervous or excitable people. The phenomena disappeared within five minutes and never seemed of a dangerous character. Only in two cases grave results supervened, and these were alarming.

One patient, a lady, was given too large a dose of adrenalin; this occurred at the beginning of our experiments; she collapsed. Her pulse was from 120 to 130; she fought for breath and had feeling of asphyxiation and intense agony; she recovered, however, within half an hour, after sulphuric ether had been administered and a cold compress applied to the heart, but she had to remain at the polyclinic for two hours under medical supervision, as the same attack came on three times when she attempted to go home.

The second patient, a young lady of twenty-two, subject to hysteria, became very excited after a normal dose had been injected. Her pulse grew rapid, but lessened after a few minutes. After the operation she was said to be completely lame in both legs and unable to walk, a result due to hysteria and certainly not to adrenalin. The above circumstances warn us to be careful in its administration, and in consequence of these experiences we have excluded all known cases of heart disease from treatment by adrenalin.

Since we reduced the dose for extraction to one milligram, no serious symptoms have been observed.

As regards secondary symptoms, they occurred only in one out of every twenty patients—a violent, in some cases, according to the statement of the patient, an almost overpowering headache, lasting from two to three and even for twenty-four hours. But it was further ascertained that they usually suffered from headache and were relieved by the use of adrenalin. Unfortunately, at that time, I did not think of these symptoms having anything to do with constitutional weakness, especially any disturbances of metabolism and of the kidneys. I did not make investigations in this direction; I intend to do so in the future.

My experiments date from October, 1902, to the beginning of June, 1903. I felt constrained to publish them through a perusal of an article by Moeller on his "Anemorenin." It was not only my desire to replace Moeller's suprarenal extract by a better and more reliable one, but also to prevent (through the publication of my results) a preparation of unknown composition from being introduced into dental practice.

Whilst my experiments were being made, Dr. H. Braun published an important work on adrenalin under the following title: "On the Influence of the Vitality of the Tissues on both Local and General Toxicity produced by Anesthetizing Agents, and on the Importance of Adrenalin for Local Anesthesia," in the *Archiv fuer Clin. Chirurgie*, 69, Nos. one and two. Unfortunately I only heard of this in May, 1903, and was very glad Dr. Braun and I had arrived simultaneously at the same conclusion with regard to the application of adrenalin for local anesthesia. *This article contains numerous experiments on animals and men, and being so clear and precise, all apprehensions as to intoxication and secondary hemorrhage and other disturbances from the administration of a normal dose of adrenalin fall to the ground.* Every dentist should read this splendid article and obtain interesting information from it. I can not conclude my remarks more appropriately on the advantages of adrenalin for local anesthesia than by quoting the words of Dr. Braun:

"*The practical importance of adrenalin for local anesthesia is not to be gainsaid; an addition of adrenalin to cocain solutions permits of diminishing their concentration and dose and partly lessens any risk of toxicity in deep anesthesia. We have never seen any bad but only good results from adrenalin. But I recommend the use of adrenalin to the surgeon not only on account of its influence on cocain anesthesia, but also because of its constrictor effect on the vessels, even to closing them. This alkaloid of Esmarch's bandage is useful in many ways, e.g., in arresting parenchymatous hemorrhage from wounds in hemophiliacs.*"

